# Evolution of an invasive ductal carcinoma to a small cell carcinoma of the breast: A case report

**DOI:** 10.1097/MD.0000000000028433

**Published:** 2022-01-14

**Authors:** Marya Hussain, Marcia Abbott, Ramin Zargham, Aliyah Pabani, Omar F. Khan

**Affiliations:** a Department of Oncology, Cumming School of Medicine, University of Calgary, Calgary, Alberta; b Department of Pathology and Laboratory Medicine, Cummings Medical School, University of Calgary, Calgary, Alberta.

**Keywords:** case report, chemotherapy, small cell, tumor heterogeneity

## Abstract

**Rationale:**

Small cell carcinoma (SCC) is a rare subtype of breast cancer and presents a complex diagnostic and treatment challenge, due to paucity of data. To the best of our knowledge, most cases of breast SCC reported in the literature describe a de novo breast primary. Our case is unique as it describes the evolution of an invasive ductal carcinoma after treatment into a SCC of the breast.

**Patient concerns and diagnosis:**

We report a case of a 53-year-old female, lifelong non-smoker, who initially presented with breast mass noted on self examination. Breast and axillary lymph node biopsy demonstrated a hormone receptor positive invasive ductal carcinoma with a metastatic T3 lesion.

**Intervention:**

She was treated with first-line palbociclib/letrozole with initial clinical response, and at progression was switched to capecitabine with no response. Repeat biopsy of the axillary lesion showed evolution of the tumor into a triple negative breast cancer. She was then treated with third-line paclitaxel and radiation therapy with good initial response. She eventually had further disease progression and presented with a new mediastinal lymphadenopathy causing SVC syndrome. Biopsy of this showed a small cell variant of breast neuroendocrine carcinoma. Due to the evolution of histology in this case, a retrospective review of her initial breast specimen as well as the second biopsy from the axilla was conducted which confirmed that the mediastinal lymphadenopathy was metastatic from the original breast tumor.

**Outcomes and lessons:**

We speculate that the initial treatment allowed a minority of treatment-resistant neuroendocrine cells to grow and become the dominant face of the tumor. Our patient had an excellent response to carboplatin/etoposide and consolidative locoregional radiotherapy but presented with an early intracranial recurrence. This is a similar pattern of metastases as seen in lung SCC and highlights a potential role for prophylactic cranial irradiation in breast SCC. Further studies are needed to better understand the biology and treatment of breast SCC which continues to present a challenge for clinicians.

## 1 Introduction

Small cell carcinoma (SCC) of the breast is rare, with an incidence reported from 0.1% to 5.0% of all breast cancers.^[1]^ SCC of the breast pathologically shares identical morphology to SCC from other sites of origin within the body. However, identification of concurrent ductal or lobular in situ or invasive carcinomas favors breast origin.^[2–9]^

Once diagnosed, tumor management guidelines for this rare histology are lacking. It also remains unclear whether to treat poorly differentiated breast carcinoma with neuroendocrine differentiation analogous to other poorly differentiated invasive breast cancers, or instead to manage them similarly to poorly differentiated neuroendocrine carcinomas of other sites.^[10]^

Nearly all cases of breast SCC reported in the literature describe a de novo breast primary. Here, we describe a unique case of a 53-year-old female who initially presented with a metastatic hormone receptor positive invasive ductal carcinoma, which evolved into SCC of the breast after treatment.

## 2 Case presentation

A 53-year-old pre-menopausal female initially noted a breast mass on self-examination. She was otherwise healthy aside from hypertension and was a lifelong non-smoker. Over the course of a month, this mass became tender with increasing pain radiating to the axilla, prompting her to seek medical attention. A subsequent mammogram and ultrasound found a right central breast mass measuring 5.9 × 3.2 × 5.4 cm with multiple satellite lesions. A biopsy confirmed a high-grade invasive ductal carcinoma, which was strongly positive for both estrogen (ER) and progesterone (PR) receptors (Allred scores 8/8 for both), and negative for HER-2 (immunohistochemistry scored as 1+). Surrounding ductal carcinoma in-situ was noted, but no lympho-vascular invasion was seen. Axillary lymph node biopsy was also positive for invasive ductal carcinoma. She was subsequently referred to medical oncology for consideration of neoadjuvant chemotherapy. Subsequent staging investigations with a CT scan of the chest, abdomen, and pelvis and bone scan showed a sclerotic bone lesion in the T3 vertebrae highly concerning for skeletal metastasis. No other site of metastatic disease was noted, specifically with no evidence of any pulmonary lesions.

She was initially treated with first line palliative palbociclib and letrozole therapy after induction of menopause with leuprolide. Bisphosphonate therapy with zoledronic acid was also initiated. Despite an initial clinical response, 6 months after starting therapy she developed increasing axillary pain, and repeat imaging showed disease progression in her breast and axilla, with stability of the T3 metastatic lesion. She was thus switched to second-line capecitabine systemic therapy.

After 3 months on capecitabine, a repeat CT scan showed significant increase in the size of 1 axillary lymph node measuring 3.3 × 2.1 cm (previously 1.6 × 1.9 cm), while showing improvement in other sites. After a review at multidisciplinary tumor boards, a recommendation was made to biopsy the axillary lesion to reassess the receptor status. This revealed metastatic carcinoma consistent with a breast primary, with similar morphology to the original biopsy. However, the biomarker status was now triple-negative (ER 0/8, PR 0/8, and HER-2 0 on immunohistochemistry).

**Figure 1:**
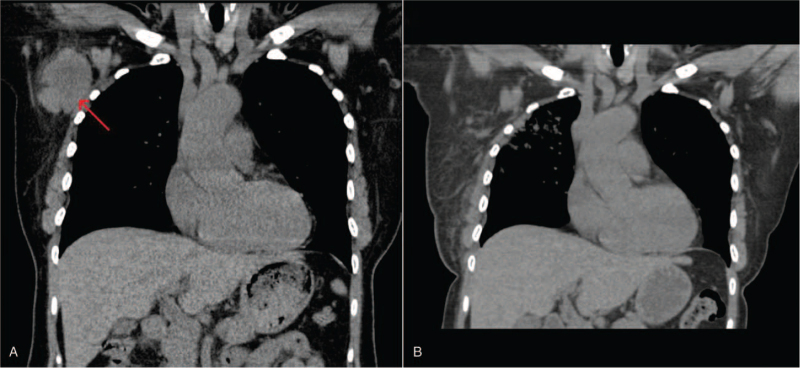
Near-complete radiographic response in right axillary lymph node conglomerate (red arrow) seen on CT scan prior to radiotherapy (A), and on subsequent CT scan 4 months after completion of right axillary radiotherapy (4500 cGy in 15 fractions) and initiation of paclitaxel chemotherapy (B).

She underwent palliative radiation therapy to the axilla/right breast (4500 cGy in 15 fractions). This was followed by third-line therapy with weekly paclitaxel. She sustained an excellent clinical and radiographic response in the axilla (Fig. 1). However, 7 months after starting paclitaxel, she presented with persistent frontal headaches, with increasing dyspnea and a non-productive cough. A workup for intracranial metastatic disease was negative, but a CT chest, abdomen, and pelvis showed new mediastinal and right hilar lymphadenopathy, as well as an enlarging opacity in the right upper lobe (which was previously reported as post-radiotherapy changes). Interestingly, the breast mass continued to shrink in size, now measuring 1.6 × 1.3 cm and her lesion at T3 remained stable. Her headaches and dyspnea were deemed secondary to superior vena cava syndrome arising from the right apical mass.

Endobronchial biopsy of the mediastinal lymphadenopathy showed a small cell variant of breast neuroendocrine carcinoma. Due to the prior axillary radiotherapy, radical dose radiotherapy could not be delivered safely. She completed 6 cycles of carboplatin and etoposide, with her post treatment PET/CT showing reduction in size of her paratracheal lymphadenopathy and right lung nodule, as well as stable size of breast mass and T3 lesion. She received consolidative radiotherapy to right lung and mediastinum (3000 cGy in 10 fractions) followed by surveillance. During treatment, she was seen at regular intervals in clinic to assess tolerability and adherence to treatment with review of regular blood work and treatment side effects. Five months into surveillance, she presented to the emergency department with ataxia and headache, and imaging revealed multiple intracranial metastatic lesions, including in the left cerebellum (2.4 × 2.7 cm) and right parietal (2.8 × 4.8 cm) regions. She was taken to the operating room due to compression of the fourth ventricle and mass effect in the posterior fossa, and subsequently underwent radiotherapy to the brain. Systemic staging investigations showed no evidence of disease progression. At the time of this report, she currently continues a surveillance approach, and is 35 months from her original breast cancer diagnosis.

### 2.1 Pathology

The endobronchial biopsy showed significant crush artifact with an exceedingly necrotic background. A dimorphic population of cells was noted, with the more predominant population consisting of small hyperchromatic irregular cells that displayed molding, atypical mitoses, and a cord growth pattern. The second population was scanter, in which the pleomorphic cells were larger with macronucleoli. The cells were mostly single, although small loosely cohesive clusters could be seen; larger clusters were intermixed with the necrotic debris. Pertinent immunohistochemistry was also completed to ascertain the origin of the malignancy. The cells were immunoreactive for synaptophysin, CK7, Cam5.2, and focal CD56; negative stains were CK20, chromogranin, ER, PR, CDX-2, Napsin, p40, GATA-3, mammaglobin, and TTF-1.

Due to the evolution of histology in this case, a retrospective review of her initial breast specimen as well as the second biopsy from the axilla was conducted. Based on cytological and immunological similarity between the specimens, it was confirmed that the mediastinal lymphadenopathy was metastatic from the original breast tumor. In the original breast biopsy, most of the tumor cells were in sheets, cords, and nests with a very small focus of necrosis; ductal carcinoma in situ was also present. Cytologically, the tumor cells were pleomorphic with enlarged hyperchromatic irregular nuclei and macronucleoli. There were numerous atypical mitotic figures and molding features suggestive. Immunostaining of the focus showed negative staining for synaptophysin and CD56 with an intermediate Ki-67 proliferative index. GATA3 and mammaglobin were positive.

**Figure 2:**
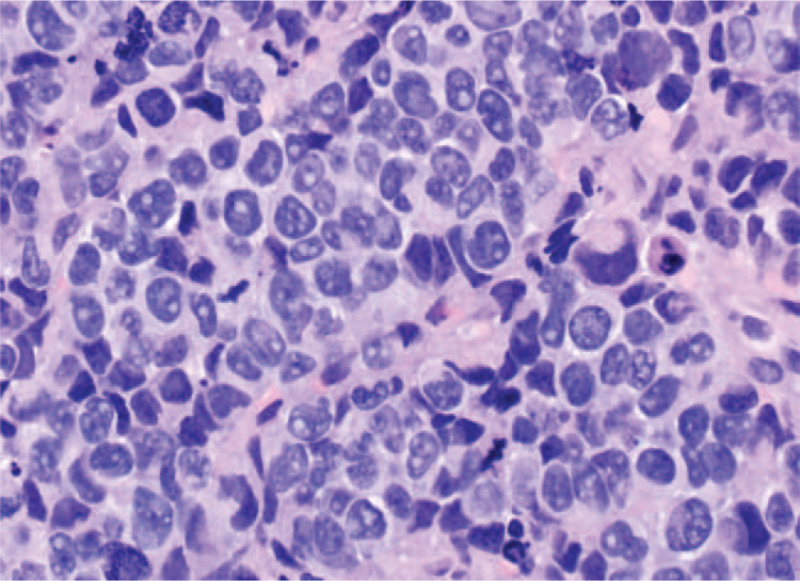
Right axillary lymph node biopsy from 2019 showing very pleomorphic cells with molding.

In the second biopsy from the axilla, the specimen consisted of similar pleomorphic cells as seen in the original breast biopsy, arranged in nests, cords, sheets, and trabeculae in an extremely necrotic background (Fig. 2). Immunohistochemical stains were performed, and the anaplastic cells were immunoreactive for AE1/AE3 and CK7, with a higher Ki-67 proliferative index and a focal area of Synaptophysin positivity. However, the tumor cells were negative for GATA3, mammaglobin, ER, PR, HER2, CK20, chromogranin, and CD56.

## 3 Discussion and literature review

Our literature search revealed 66 English language case reports,^[1–8,11–69]^ describing the clinical course of 91 patients with poorly differentiated neuroendocrine breast cancer (Supplemental Digital Content Table S1). The first case was reported by Wade et al . Most cases are of de novo small cell carcinoma of the breast, whereas our case is unique as it describes the evolution of an invasive ductal carcinoma after treatment into a SCC of the breast.

The patient and tumor characteristics at diagnosis are described in Table 1. We note that, while patients were mostly females, 4 cases were reported in males (a higher proportion than would typically be expected in breast cancer).^[17,36,44,58]^ Interestingly, more patients were found to be non-smokers, in contrast to what would be commonly seen in lung SCC.^[3,4,8,14,27,38,43,68]^

**Table 1:** Patient demographic and tumor characteristics based on 66 previous case reports.

Patient characteristics	
Age (yrs)	
Mean	54
Range	25–83
	**n (%)**
Gender	
Male	4 (4.7)
Female	82 (95.3)
Missing data	5
Menopausal status	
Pre	10 (30.3)
Post	23 (69.7)
Missing data	54
Smoker	
Yes	2 (20)
No	8 (80)
Missing data	81

Tumor characteristics	
Size (cm)	
Mean	4
Range	1–14.5
	**n (%)**
Stage	
Non metastatic	67 (90.5)
Metastatic^∗^	7 (9.5)
Missing data	17

∗ Sites of metastases included liver, bone, lymph nodes, serosa, and lymphangitic carcinomatosis.

There are no unique imaging characteristics that help differentiate SCC from invasive ductal carcinomas in the breast. Guo et al discussed detection of breast SCC based on imaging features, but found that even on MRI it was very difficult to differentiate SCC from other triple-negative breast cancers.^[69]^

Many authors describe the absence of a lung primary on initial staging investigations as one of the major factors in determining the primary site of origin of SCC.^[12,16,48,70]^ In our case, this posed an additional dilemma as the site of new tumor growth was in the lung itself, with a lung mass and mediastinal lymphadenopathy. Hence, in cases such as ours, where a lung mass is present as well, further ancillary testing on pathology specimens can support the diagnosis of SCC of breast.

## 4 Pathologic features

In retrospect, the breast tumor first showed a focal area of neuroendocrine features (without positive ER or neuroendocrine staining) while most tumor cells were ER, PR, mammaglobin and GATA3 positive. However, after treatment the subsequent biopsies from the axilla and from the paratracheal lymphadenopathy showed trends towards positivity for neuroendocrine markers, negativity for breast markers and higher Ki-67. Most of the cases described in the literature report a high Ki-67 of >20%.^[1,12,14–16,31,33,42,46,48,49,68,69]^

TTF-1 was negative in our case, supporting the diagnosis of a non-pulmonary SCC, as 90% of pulmonary SCC express this marker.^[71]^ Interestingly however, TTF-1 has been reported positive in a few cases of breast SCC, so this again cannot be used as a reliable indicator to determine origin.^[14,16,27,28,45,50,68]^ Breast SCC does not appear to have a characteristic pattern of neuroendocrine staining on pathology, with heterogeneity noted in the literature.^[5,6,21,70]^ Given the characteristic morphology of SCC, multiple authors report that negative neuroendocrine marker staining does not preclude the diagnosis of breast SCC.^[19,35]^ Breast SCC can express ER and/or PR positivity, with reported rates ranging between 25% and 73%.^[26,72]^ However, to our knowledge, no cases of HER-2 positive breast SCC have been reported.^[8,26,70]^

Genomic mutational analysis may also help differentiate breast SCC from other primary SCC sites. One study retrospectively identified cases of SCC of breast and lung. Next generation sequencing found a statistically significant difference in the expression of PIK3CA mutations; 33% of breast SCCs expressed PIK3CA mutations, while no lung SCCs expressed PIK3CA mutations (*P* = 0.005). Neither group expressed PD-L1, while approximately half of both groups expressed PD-1.^[73]^ A case report by Niravath et al describes a lifelong non-smoker, with a BRCA2 mutation who initially had an invasive ductal carcinoma treated with neoadjuvant chemotherapy with complete pathologic response. Her tumor subsequently recurred, with SCC found on pathology. It was deemed to be a recurrence of the same tumor based on genomic analysis showing presence of identical PIK3CA mutations in both tumors.^[14]^

Temporal heterogeneity is particularly important when comparing the features of the primary tumor and metastatic or recurrent lesions, and preinvasive and invasive disease in the same tumors.^[74]^ Our case highlights both spatial and temporal heterogeneity, with the SCC clone only identified on a third biopsy. This underscores that therapy can eliminate dominant clones, selecting for rare but resistant ones.^[14]^ As a result, it is critical to consider obtaining new tissue samples in cases of disease progression.

## 5 Management and prognosis

Management of de novo breast SCC is challenging given its rarity. In cases such as ours where intra-tumoural heterogeneity exists, treatment decisions become increasingly complicated. Variable treatment regimens have been trialed in cases of de novo breast SCC. Most commonly, treatment is extrapolated from lung SCC, with 4–6 cycles of carboplatin/etoposide or cisplatin/etoposide^[16,23,26]^ either in the neoadjuvant or adjuvant setting used.

Three of the 7 metastatic cases reported in literature treated patients with platinum/etoposide and noted some initial radiologic or clinical response but variable overall survival, ranging from 6 to 12 months with an average of 10 months.^[1,23]^ One metastatic ER/PR positive case was treated with endocrine therapy alone, with complete response and the patient remained recurrence free for at least 12 months after treatment.^[75]^ Another metastatic breast SCC case described by Wade et al was treated with doxorubicin/vincristine/cyclophosphamide with no response, followed by rapid disease progression.^[43]^

In contrast to pulmonary SCC, curative-intent surgery should be considered where possible in breast SCC patients.^[23]^ For early stage or locally advanced breast SCC, numerous systemic therapy regimens have been utilized. These include 5-fluorouracil, doxorubicin, and cyclophosphamide followed by paclitaxel and etoposide (with a partial response,^[31]^ irinotecan and carboplatin followed by radiotherapy (with a complete response),^[32]^ docetaxel and cyclophosphamide (with a partial pathologic response),^[15]^ and 5-fluorouracil, epirubicin and cyclophosphamide (with no pathologic response).^[26]^ Some cases were treated with up-front mastectomy and axillary lymph node dissection, with or without adjuvant treatment. Overall survival was highly variable (average 28 months, with a range of 1–96 months) depending on size of the tumor and lymph node status.

After completion of treatment, the most common sites of recurrence were lung, bone, liver, lymph nodes (parasternal, mediastinal, subcarinal and neck) and brain. Less common sites of recurrence included chest wall, localized skin, ipsilateral breast, and thyroid. Our case also highlights the importance of monitoring for intracranial involvement of breast SCC. Of note, prophylactic cranial irradiation (PCI) was not pursued in our case, but our patient developed multiple intracranial metastatic lesions despite ongoing systemic disease response to chemoradiation. The role of PCI in extrapulmonary SCC is controversial, although one study noted that PCI was associated with improved prognosis.^[76]^ Therefore, we recommend multidisciplinary discussion regarding consideration of PCI (and of MRI surveillance for intracranial metastatic disease if PCI is not pursued) in locally advanced or metastatic breast SCC patients, consistent with pulmonary SCC guidelines.^[77]^

Several studies have been conducted to analyze the epidemiology and prognostic factors and patterns of care for extrapulmonary SCC. One study conducted on 4397 patients in the United States between 1975–2016, demonstrated that breast SCC comprised about 5.0% of all extrapulmonary SCC cases. In this study, prognosis was varied depending on tumor site, but breast SCC showed the highest 5-year survival (42.0%).^[78]^ This was re-demonstrated in a European study in 2020, with breast SCC having a median survival of 3.05 years. The study also found that chemoradiation was associated with improved overall survival when compared to radiotherapy alone, chemotherapy alone, and “no treatment” regardless of anatomic subsite or stage. Unlike other sites such as genitourinary SCC, a substantial survival improvement in breast SCC was seen with surgery.^[79]^

## 6 Conclusion

The diagnosis and management of breast SCC presents a multi-level challenge for oncologists and pathologists. We highlight the importance of considering spatial and temporal heterogeneity within breast cancers. This phenomenon explains the lack of response to initial treatment seen in our case. Although it is difficult to prove, we speculate that treatment of the invasive ductal component allowed a minority of treatment-resistant neuroendocrine cells to grow and become the dominant face of the tumor.

Tumor stage remains the most important prognostic factor for breast SCC, and surgical resection should be considered where feasible. SCC of breast appears to respond to treatment with a platinum/etoposide combination like its lung counterpart, although the degree and duration of responses is variable. Our case demonstrated an excellent systemic response to carboplatin and etoposide, followed by locoregional radiotherapy. However, it also highlights the potential for intracranial recurrence, and we suggest considering PCI in locally advanced or metastatic breast SCC patients. Further studies regarding predictive and prognostic biomarkers in breast SCC (including PIK3CA and PD-1 expression) are needed.

## Author contributions

**Conceptualization:** Aliyah Pabani, Omar F. Khan.

**Data curation:** Marya Hussain.

**Supervision:** Ramin Zargham, Aliyah Pabani, Omar F. Khan.

**Writing – original draft:** Marya Hussain.

**Writing – review & editing:** Marya Hussain, Marcia Abbott, Aliyah Pabani, Omar F. Khan.

## Supplementary Material

Supplemental Digital Content
